# Diarrhœa

**Published:** 1866-08

**Authors:** 


### DIARRHŒA

Is a very common disease in Summer-time. Cholera is nothing more than exaggerated diarrhœa. When a man has died of diarrhœa, he has died of cholera, in reality. It may be well for travellers to know, that the first, the most important, and the most indispensable item in the arrest and cure of looseness of the bowels, is absolute quietude on a bed; Nature herself always prompts this, by disinclining us to locomotion. The next thing is, to eat nothing but common rice, parched like coffee, and then boiled, and taken with a little salt and butter. Drink little or no liquid of any kind. Bits of ice may be eaten and swallowed at will. Every step taken in diarrhœa, every spoonful of liquid, only aggravate the disease. If locomotion is compulsory, the misfortune of the necessity may be lessened, by having a stout piece of woollen flannel bound tightly round the abdomen, so as to be doubled in front, and kept well in its place. In the practice of many years, we have never failed to notice a gratifying result to follow these observances.

